# An Improved Method for Magnetic Nanocarrier Drug Delivery across the Cell Membrane

**DOI:** 10.3390/s18020381

**Published:** 2018-01-29

**Authors:** Behzad Mehrafrooz, Maysam Z. Pedram, Ebrahim Ghafar-Zadeh

**Affiliations:** 1 Department of Mechanical Engineering, K.N. Toosi University of Technology, Tehran 16315-1355, Iran; behzadmehrafrooz@gmail.com; 2 Faculty of Electrical and Computer Engineering, K.N. Toosi University of Technology, Tehran 16315-1355, Iran; 3 Departement of Electrical Engineering and Computer Science, York University, Toronto, ON M3J1P3, Canada

**Keywords:** magnetic nanocarrier, magnetic field, molecular dynamics simulations, drug delivery, magnetic resonance imaging (MRI)

## Abstract

One of the crucial issues in the pharmacological field is developing new drug delivery systems. The main concern is to develop new methods for improving the drug delivery efficiencies such as low disruptions, precise control of the target of delivery and drug sustainability. Nowadays, there are many various methods for drug delivery systems. Carbon-based nanocarriers are a new efficient tool for translocating drug into the defined area or cells inside the body. These nanocarriers can be functionalized with proteins, peptides and used to transport their freight to cells or defined areas. Since functionalized carbon-based nanocarriers show low toxicity and high biocompatibility, they are used in many nanobiotechnology fields. In this study, different shapes of nanocarrier are investigated, and the suitable magnetic field, which is applied using MRI for the delivery of the nanocarrier, is proposed. In this research, based on the force required to cross the membrane and MD simulations, the optimal magnetic field profile is designed. This optimal magnetic force field is derived from the mathematical model of the system and magnetic particle dynamics inside the nanocarrier. The results of this paper illustrate the effects of the nanocarrier’s shapes on the percentage of success in crossing the membrane and the optimal required magnetic field.

## 1. Introduction

In the last few years, there has been a growing interest in discovering treatments for diseases by using magnetic cell membrane injection. In general, the lipid bilayer is a fundamental component of all biological cells and provides their universal structure. It plays a critical role in the passage and blockage of substances into the cell [1]. Modern drug targeting delivery technologies have been proposed to overcome these cellular membranes. Regarding the significant advances in nanotechnology and biotechnology, nanoscale structures are extensively used in cellular biology. A widely-used method in this scope is exploiting nanocarriers such as carbon nanotubes (CNT) and nanocapsules, i.e., fullerene. Generally, carbon-based nanocapsules have been proposed as drug carriers, which may be used to realize the magic bullet concept suggested by Paul Ehlrich [2], which refers to the capability of targeting a drug carrier to a particular site and releasing its contents when desired. Thus, many papers have been conducted investigating the interaction of carbon-based nanocarriers and the cell membrane. As nanoinjection of nanocapsules requires exerting external forces, recently, magnetic nanocapsules (MNCs) were proposed to serve as a remarkable strategy to provide these external forces [3,4]. By using the magnetic resonance imaging (MRI) device, Martel et al. [5,6] reported a novel technology for a therapeutic magnetic microcarrier. Nacev et al. [7] presented an analysis of nanoparticle distribution, in and around a single blood vessel located at any depth in the body, with any physiologically-relevant blood flow velocity, diffusion and extravasation properties and with any applied magnetic force on the particles. Moreover, Using physical first-principles and a sophisticated vessel-membrane-tissue (VMT) numerical solver, Nacev et al. [8] comprehensively analyzed in detail the behavior of magnetic particles in blood vessels and surrounding tissue.

The concept of magnetic nanoparticles in drug delivery systems has been widely investigated over the past few decades. So far, several studies have been conducted in this regard [9,10,11,12]. Katagiri et al. [13] studied magnetoresponsive smart capsules formed with polyelectrolytes, lipid bilayers and magnetic nanoparticles. Using MRI technology, the development of functionalized superparamagnetic iron oxide nanoparticles with a PEG-modified, phospholipid micelle coating and their delivery into living cells have been reported in [14]. The MRI system also has been used in several targeted drug delivery systems to different organs and tissues, such as gliomas [15], the cardiovascular system [16] and tumor tissues [17]. The ability to use magnets external to the body to focus therapy to deep tissue targets has remained an elusive goal in magnetic drug targeting [18]. Generally, clinical methods are highly expensive and also are based on trial and error. Therefore, magnetic-based drug delivery approaches may not be practical and affordable in all situations, and accordingly, exploiting computational methods to predict the efficiency of this method may reduce the need for costly and expensive experiments.

Nowadays, computational simulations have become an attractive means because of their low cost and inherent speed [19]. Several types of research have been conducted on this new technology to investigate the different aspects of the drug or gene delivery to the cells, and different methods, i.e., molecular dynamics (MD), dissipative particle dynamics (DPD) and coarse-grained MD (CGMD) simulations, have been employed to explore the interaction between nanocarriers and the lipid bilayer. By experiments, the mechanism of CNT penetration of the plasma membrane has been explored [20,21]. Furthermore, CNTs have been studied in drug delivery by conjugating them non-covalently or covalently with drugs, biomolecules and nanoparticles [22]. MD simulations comprise another method that is widely employed. In [23], a series of MD simulations was conducted to investigate the interactions of single-walled (SW) and multi-walled (MW) CNTs with a lipid bilayer membrane. More precisely, this study figured out the effects of the size and type of CNT on the mechanism of spontaneous exothermic insertion of CNTs into the lipid bilayer membranes. MD simulations have been employed to examine the penetration of a CNT into a pure 1-palmitoyl-2-oleoyl-sn-glycero-3-phosphocholine (POPC) cell membrane under various injection velocities, CNT tilt angles and chirality parameters [24]. Raczyński et al. [25] demonstrated a series of steered molecular dynamics (SMD) simulations on two types of armchair CNTs, open-ended and capped, to examine the process of nanoindentation of the phospholipid bilayer by SWCNTs. Moreover, in vitro studies showed that nanorods can improve the efficacy of antimicrobial amphotericin B treatments [26]. Other carbon-based nanoparticles in drug delivery systems are fullerenes. Atomistic molecular dynamics simulations of a C60 fullerene in a fully-hydrated dimyristoylphosphatidylcholine (DMPC) lipid membrane have been conducted [27]. In this research, the interaction with and passive transport into lipid membranes of fullerenes have been explored. The works in [28,29] demonstrated atomistic MD simulations of C60 fullerenes inside a DMPC lipid bilayer.

This study aims to scrutinize the characteristics of the appropriate magnetic field that is required to push a nanocarrier through a cell membrane. We will examine the passage of coated nanocarriers through a lipid bilayer in the drug delivery process. We have to coat these nanocarriers with protein chains in order to guarantee their safe passage through the cell membrane. In other words, the bonds forming between these chains and the cell membrane receptors may reduce the severity of cell membrane damage. The main novelty of this paper is determining the proper magnetic field for the nanocarriers to cross the membrane. The literature reports essentially that previous studies have reported a constant force field, but none of them have focused on finding the optimal magnetic field. Furthermore, few papers have been conducted on the shape effects of the nanocarrier. Moreover, this paper studies and compares the penetration of an end-capped CNT and fullerene into a pure cell membrane under various injection velocities, with more emphasis on finding the proper required magnetic field.

The remainder of the paper is as follows: Section 2 presents the methodology of the paper. Section 3 illustrates the nanocarrier types and design properties. Moreover, MD simulations of the penetration of these nanocarriers into the cell membrane are illustrated in Section 3. Section 4 reports the system identification and the design of the optimal magnetic field for the nanocarrier to cross. Finally, the paper concludes with some remarks and future work.

## 2. Methodology

**Figure 1:**
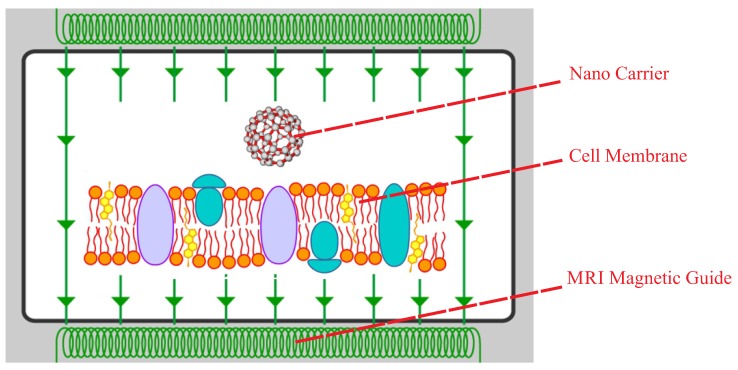
Schematic model of the nanocarrier passing through the membrane using the MRI device.

**Figure 2:**
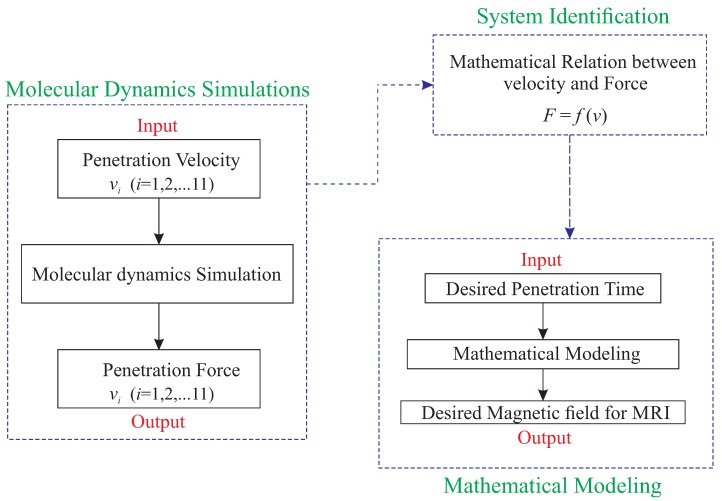
Schematic diagram of methodology of modeling the indentation of the nanocarrier through the membrane using MRI device.

In this paper, the main objective is to find the proper magnetic field for the crossing of the nanocarrier in the desired time as illustrated in Figure 1. To do so, first, the all-atom molecular model of the system is simulated via molecular dynamics modeling. As previously mentioned, two different nanocarriers, namely end-capped CNT and fullerene, are modeled. Then, using steered molecular dynamics simulation, the required force for constant velocity penetration of the nanocarrier is extracted. Due to the huge computational costs associated with molecular dynamics modeling techniques, the system identification method is employed: a mathematical function between the penetration velocity and required force is derived. In other words, in order to prevent modeling of penetration for a high range of velocities, a mathematical function is derived by using the system identification of a series of penetrations simulated by molecular dynamics. In the final step of this paper, the proper magnetic field is found for the penetration of the nanocarrier. In this step, the magnetic field and its gradient for the MRI device are extracted by applying the limits of the MRI device. Figure 2 represents the schematic diagram of the methodology of this paper and its three main steps.

## 3. Materials and Methods

**Figure 3:**
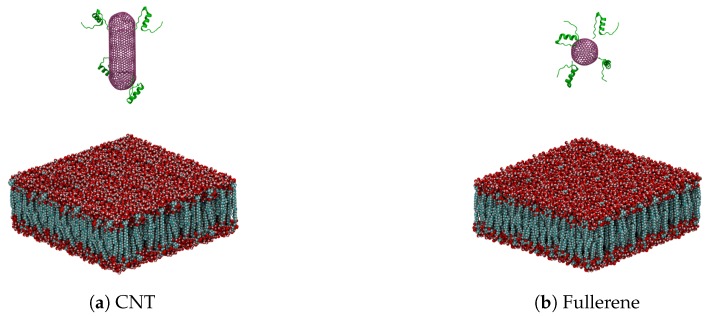
Atomic representation of two simulated systems (lipid bilayer atoms represented with their van der Waals radius; nanocarrier (fullerene and end-capped CNT) atoms are rendered with line representation (purple), and insulin chains are represented in cartoon).

As mentioned earlier, a typical approach applied in several MD simulations in the literature is using a carbon fullerene as a nanocarrier for site-specific drug delivery. Hence, it has been used in this research. The C720 fullerene, which is the biggest fullerene with an available PDB file, is selected as the first type of nanocarrier that we use. Another approach for producing a bigger nanocarrier is to use CNTs, because the end-capped nanotube is less invasive compared to the open-ended one [25]. Therefore, the most desirable model is to use the biggest possible fullerene, which is C720, as the end-cap of a bigger nanotube. Although this method is desirable theoretically, its performance has several limits. Generally, a nanotube is specified by two numbers (*m*, *n*) and another number as its length. The relationship between the diameter of a cylinder and (*m*, *n*) is as below:

(1) $$d=\frac{a_{0}}{\pi }\sqrt{n^{2}+m^{2}+nm}$$

in which $a_{0}$ is the lattice constant and is equal to $a_{0}=0.246$ nm. C720 has a diameter of around 2.4125 nm. In order to satisfy the relation mentioned above for the nanotube diameter, we found the best set of (*m*, *n*) integers equal to (15, 20) subsequently. Therefore, the diameter of the nanotube is about to 2.38 nm, which is slightly smaller than the diameter of C720. Hence, we select six atoms of each section of C720 and their nearest six atoms of the nanotube at both ends, and in total, we define twelve new covalent bonds in order to bond the end-caps to the nanotube. In the next step, it is desired to attach the insulin chains to the surface of the nanocapsule. The presence of these chains and the bonds hat form between these chains and the receptors of the cell membrane provide a safe situation for the passage of the nanocapsule through the cell membrane. In this research, by considering the relative size of the insulin chain and nanocapsule, we have selected four similar chains in order to attach them to each nanocapsule. The relative position of the chains and nanocapsule was adjusted to have a carbon atom of each insulin near a carbon atom of the nanocapsule. For a complete bond between the chains and nanocapsule, a covalent bond between a carbon of each insulin with the nearest carbon of the nanocapsules is defined. Figure 3a,b illustrates two types of nanocapsules with attached insulin chains.

All-atom MD simulations are used to identify the nanocapsule penetration process with NAnoscale Molecular Dynamics (NAMD) [30], a parallel molecular dynamics code designed for high-performance simulation of large biomolecular systems implementing the popular CHARMM27 force field [31]. The most important part of MD simulation is the calculation of the atomic interactions, which are defined by force fields. These force fields describe the complete behavior of the nanocarrier and lipid bilayer. The CHARMM27 force field is based on harmonic and anharmonic terms describing the potential of bonds, angles and dihedrals. The non-bonded interactions are modeled by a Lennard–Jones 6-12 potential and by the ionic (Coulomb) interactions. Moreover, the Visual Molecular Dynamics (VMD) [32] was employed for simulation setups, visualization and trajectory analysis. All simulations were carried out with a four-core XEON (X5650, 2.67 GHz) server computer.

All of the simulations consist of a nanocapsule and a POPC lipid bilayer with initial dimensions of 130×130×55 ${Å}^{3}$. Because in reality, penetration of the lipid bilayers takes place in an environment with enough water molecules, we simulated 7784 water molecules near the hydrophilic heads of the lipid bilayer, which consist of 63,114 atoms, so in total, the cell membrane is modeled by 86,466 atoms. All simulations are conducted in the isothermal–isobaric (constant total number of particles (*N*), pressure (*p*) and constant temperature (*T*-NpT) ensemble using three-dimensional periodic boundary conditions, and the time step in all of the simulations is set to 1 fs. The water molecules are described using the TIP3P model. Using a Langevin thermostat with the damping coefficient equal to 1.0 ps${}^{-1}$, the temperature is controlled to be constant at 310 K during the simulation. Moreover, a Nosé–Ho0ver barostat is applied to set the pressure to 1 bar. The same conditions are considered for SMD simulations. The distance between the center of the nanocapsule and the center of the lipid bilayer membrane is set to 7 nm in each study case. A cutoff of 12 Å was used for Lennard-Jones (excluding scaled 1–4) interactions, which were smoothly switched off between 10 Å nm and 12 Å. and the neighbor list, used for speeding up the calculation of the non-bonded interactions, was kept to 14 Å. Moreover, it is noteworthy to mention that The effect of the magnetic field is simulated through the modified velocity Verlet algorithm [33] coupled to NAMD2. Table 1 presents the properties of two types of nanocapsules.

In this part, the constant velocity pulling method is applied to push two types of nanocapsules with ten different velocities downward through the lipid bilayer in order to probe the effects of the injection velocity and nanocapsule shape. Eleven different pulling velocities were studied: 1.0 Å/ps–2.0 Å/ps with a 0.1 interval. All carbon atoms in the nanocapsule, except insulin chains, are considered as a group, and by means of SMD, constant velocity is applied to it. Force constant *k* was set to 7 kcal/mol/${Å}^{2}$. The RMSD of the nanocapsule, the temperature of the system and, finally, the force exerted on the nanocapsule were observed thoroughly during this simulation. The simulation of the penetration of the lipid bilayer by two types of nanocapsules, namely end-capped CNT and fullerene, has been conducted for eleven different pulling velocities. The simulation of the penetration of the lipid bilayer by two types of nanocapsules, namely end-capped CNT and fullerene, has been conducted for 11 different pulling velocities.

**Figure 4:**
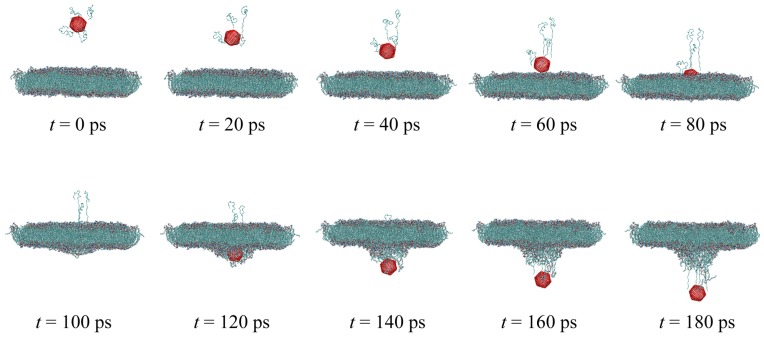
Snapshots of the cell membrane penetration by the fullerene nanocarrier with pulling velocity *v* = 1.0 Å/ps for ten penetration depths (the lipid bilayer is depicted in Van der Waals representation; fullerene is colored red; and the cartoon representation is employed for insulin chains).

**Figure 5:**
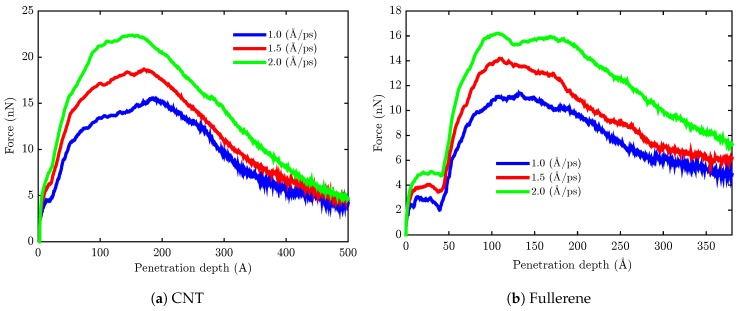
The plot of the average force required for pulling the rod nanocarrier through the bilayer, versus the penetration depth at different velocities.

**Figure 6:**
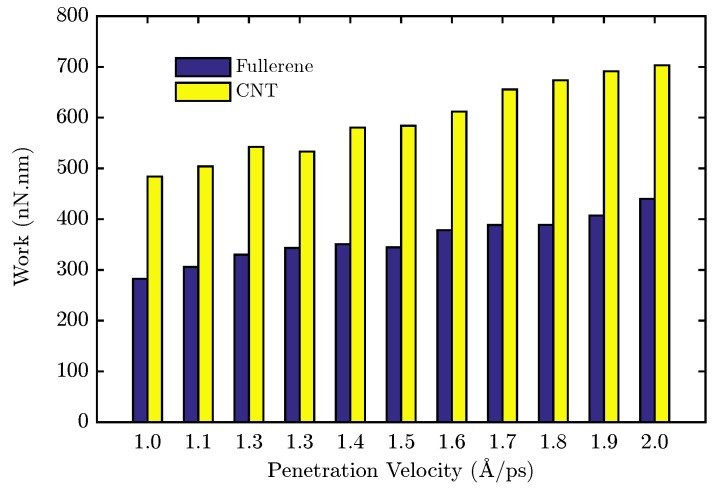
Work *w* required to pull fullerene and end-capped CNT through the cell membrane, for various pulling velocities.

**Figure 7:**
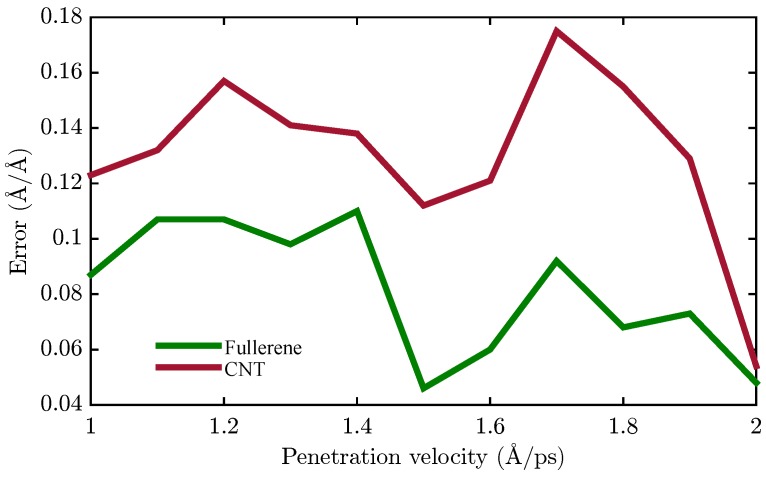
RMSD values of lipid bilayer atoms in the model for two different types of nanocarriers.

Simulation results can be better understood by referring to Figure 4. The latter figure shows the sequential representation of fullerene penetration through the cell membrane with pulling velocity v=1.0 Å/ps. It can be seen from Figure 4 that the penetration of the nanocarrier causes a slight deflection of the phospholipid bilayer during the nanoindentation process. The total force *F* required to exert on the nanocapsule in order to penetrate the lipid bilayer was calculated with respect to the penetration depth *d* and is presented in Figure 5 for each pulling velocity. For the sake of better illustration, in this figure, only 3 of 11 velocities are depicted. From the data in Figure 5, it is apparent that increasing the penetration velocity leads to the increase of the required pulling force *F* for both of the nanocarriers. This trend could be attributed to greater bending of the bilayer surface at a lower speed of penetration. What is surprising is that there are significant changes between the penetration force of fullerene and end-capped CNT. The maximal value of the force required to push the end-capped CNT is higher than that of fullerene. In order to have a better comparison of the penetration of fullerene and end-capped CNT, the work performed when the nanocarrier was pushed through the membrane is calculated using the following equation:

(2) $$W\left(d\right)=\int_{d_{0}}^{d}F\left(z\right)\;dz$$

where *d* is the depth of penetration and *z* represents the displacement of the nanocarrier along the penetration axis. The calculated work for each velocity in the two types of nanocapsules is illustrated in Figure 6. As is apparent, the required work increased by increasing the velocity, and due to the size of the end-capped CNT, the necessary work is greater than the fullerene, which is an expected result. In the next step, the RMSD method is employed to compare the destruction of the lipid bilayer with respect to the two different nanocarrier types. In fact, RMSD is a criterion that illustrates the average displacement of the total atoms relative to their initial conditions. In the following, the RMSD of the membrane’s carbon atoms is calculated versus the depth of penetration instead of time, because of the different velocities in the different cases. Now, the slope of the RMSD versus the depth of penetration is studied. In fact, a constant and high slope is a sign of the successful penetration across the membrane because the constantly increasing RMSD values of the membrane carbons are the result of them steadily being pushed away by the nanocapsule. In order to have a precise tool to compare each velocity in the two types of nanocapsules, a trend line is fitted to the results of the simulation. The slope of the line for two types of nanocarrier is plotted in Figure 7. For each velocity, the slope finds bigger values. In both cases, by increasing the pulling velocity, the slope and the error of the trend line consequently increase, while a higher range of destruction in the lipid bilayer is observed for the end-capped CNT compared to the case of fullerene.

## 4. Mathematical Modeling and Identification of the System

System identification uses algorithms to express systems in mathematical equation format in order to specify the dynamic behavior of a system in either the time or frequency domain. It is also not restricted to unique systems, and it includes industrial processes, economic and even biological systems. There are globally two strategies in system identification: gray box and black box. The gray box approach of modeling is based on physical knowledge with data aggregated from the system; physical experience gives structure to the mathematics equation with a few unknown parameters [34].

These parameters have to be estimated by identification methods. The black box approach is not based on a model, and most of the algorithms are categorized into this type. In the current study, we do not have any information about the dynamics of the membrane, so our approach is the black box type.

### 4.1. Identification Methodology

In the present research, we used the prediction error minimization (PEM) approach to estimate the coefficients. These coefficients are located by the system transfer function. In general, the estimation methods are conducted in two steps in order to finalize the parameters:

1. Initializing the parameters:
  In this section, the closest value to the parameters has to be selected according to an initial guess. Selecting the parameters could be based on physics behavior of the system.
2. Updating the parameters:
  In this section, the parameters have to be changed to track the desired value over time in iterative steps.

Estimation is separated into two domains: the continuous-time domain and the discrete-time domain. This study is conducted in the discrete-time domain because the structure of the molecular dynamics simulation has been modeled in the discrete-time domain. Therefore, considering the system behavior in this domain may cause the results to approach a precise prediction. PEM exercises numerical optimization to minimize the cost function, a weighted norm of the prediction error, defined as below for scalar outputs:

(3) $$V_{N}\left(G,H\right)=\sum_{t=1}^{N}e^{2}\left(t\right)$$

where e(t) is the difference between the measured output and the predicted output of the model. For a linear model, the error is defined as:

(4) $$e\left(t\right)=H^{-1}\left(q\right)\left[y\left(t\right)-G\left(q\right)u\left(t\right)\right]$$

where e(t) is a vector and the cost function $V_{N}\left(G,H\right)$ is a scalar value. *N* indicates that the cost function is a function of limited data samples, and estimation would be more accurate if the *N* would tend to be larger. The algorithm minimizes the prediction error to specify the transfer function coefficients that are minimizing the prediction error. In Equation (4), polynomials G(q) and H(q) have unknown values, and the coefficient of this polynomial is obtained through the order of the system.

### 4.2. Membrane System Identification

Initially, we have to excite the system by suitable input time series data to determine the identification. In this case, the velocity of the nanoparticle is fixed based on the MD simulation. As a result, the particle membrane force is extracted. The velocity of the nanoparticle varies between 1.0 Å/ps and 2.0 Å/ps.

**Figure 8:**
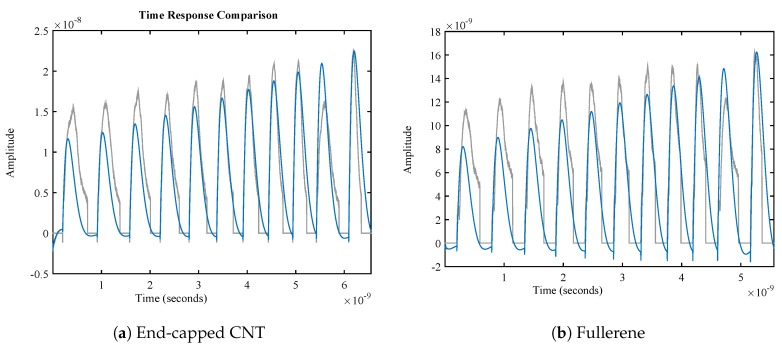
Best fitness for system identification. This figure shows the fitness quality of the data, which has been recorded from the molecular-level simulation. In this figure, diagrams of both the molecular level force and the mathematical model are illustrated.

In this identification, the input to the system is impulses, denoting the amplitude of the velocity. This velocity is exactly equal to the velocity at which the nanoparticle crossed through the membrane. The order of the transfer function would be gathered from the best fit to the data. In this identification, the best fitness of 74% with MSE was assessed to be almost $4.88\times 10^{-17}$. The transfer function is expressed in Equation (5). Figure 8 shows the best fitness of the identified transfer function for a few sampled data. This function has the general form of:

(5) $$F\left(z\right)=\frac{\left(1-bz^{-1}\right)\left(1+cz^{-1}+dz^{-2}\right)\left(1-ez^{-1}+fz^{-2}\right)}{\left(1-gz^{-1}+hz^{-2}\right)\left(1+iz^{-1}+jz^{-2}\right)\left(1-kz^{-1}+lz^{-2}\right)}\;\;a\;\;v\left(z\right)$$

where in Equation (5), *v* is nanoparticle velocity and *F* is the total force exerted on the nanoparticle. Moreover, a,b,…,l are constant parameters, which are provided for each case of the nanocarrier in Table 2.

**Figure 9:**
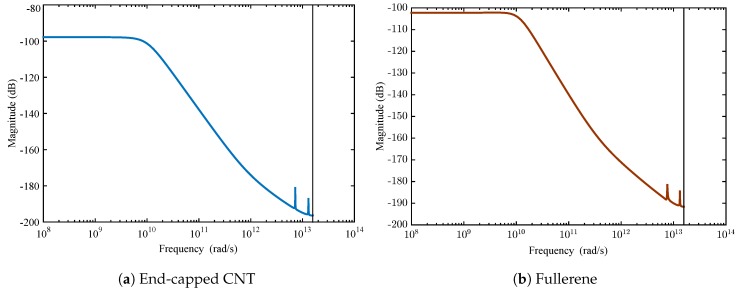
Bode diagram for the identified system.

Besides the time response of the system, the frequency domain has many data to be analyzed. This represents the variation of the system dynamics through the frequencies much better. A suitable transfer function is evoked by the MATLAB identification toolbox and setting the arranged input-output data as an identification data (iddata) object. Figure 9 displays the Bode diagram of the entire dataset.

As observed in Figure 9, the Bode diagram of the input-output system data holds a region in the frequency domain, which has a constant gain. In fact, the linear model at a frequency less than 1013 Hz can be satisfied with further qualifications. Regarding the system behavior, uncertainty needs to be added at a higher frequency in order to compensate the nonlinearity in the system.

### 4.3. Design of the Optimal Magnetic Field for Crossing the Membrane

This section discusses the system model achieved by the previous section. Thus, we are going to calculate the optimal magnetic field in order to create the suitable force and then move the nanoparticle through the membrane at a suitable velocity. In fact, the duration and velocity of magnetic nanoparticles crossing through the membrane are achieved by the mathematical model of the system. For this purpose, the force applied to magnetic nanoparticles is derived in Equation (6). Equation (12) is obtained as the result of expanding Equation (6) and confining it to one direction. Delimiting the velocity needed for crossing the membrane contributes to the calculation of the magnetic field’s profile. Equation (6) reveals the magnetic force acting on a magnetic dipole, in an applied magnetic field [35]:

(6) $$\vec{F}=\left(\vec{m}\cdot \nabla \right)\vec{B}$$

For the magnetic susceptibility of the bead, the conventional formula for the force acting on a superparamagnetic bead in the magnetic field is Equation (7) [35].

(7) $$\vec{F}=\frac{V\chi_{bead}}{\mu_{0}}\left(\vec{B}\cdot \nabla \right)\vec{B}$$

Equation (4) can be expanded as Equation (8),

(8) $$\left(\vec{B}\cdot \nabla \right)\vec{B}=\begin{array}{c} {\hat{e}}_{x}\left(b_{x}\frac{\partial b_{x}}{\partial x}+b_{y}\frac{\partial b_{x}}{\partial y}+b_{z}\frac{\partial b_{x}}{\partial z}\right)\\ +{\hat{e}}_{y}\left(b_{x}\frac{\partial b_{y}}{\partial x}+b_{y}\frac{\partial b_{y}}{\partial y}+b_{z}\frac{\partial b_{y}}{\partial z}\right)\\ +{\hat{e}}_{z}\left(b_{x}\frac{\partial b_{z}}{\partial x}+b_{y}\frac{\partial b_{z}}{\partial y}+b_{z}\frac{\partial b_{z}}{\partial z}\right) \end{array}$$

The force required for crossing is perpendicular to the plane, and the effective force has to be parallel to the normal vector of the plane of the membrane. As a whole, Equation (8) specifies that the third element must be nonzero. By selecting $b_{x}=b_{y}=0$, the magnetic force computation is simplified to Equation (9):

(9) $$\vec{F}=\frac{V\chi_{bead}}{\mu_{0}}\left(b_{z}\frac{\partial b_{z}}{\partial z}\right){\hat{e}}_{z}$$

Suppose that $Y\left(z,t\right)=b_{z}$ and by computed force $F=F_{z}e_{z}$, then:

(10) $$F_{z}\left(t\right){\hat{e}}_{z}=\frac{V\chi_{bead}}{\mu_{0}}\left(b_{z}\frac{\partial b_{z}}{\partial z}\right){\hat{e}}_{z}$$

Moreover, using the new definition in Equation (11):

(11) $$U\left(t\right)=\frac{F_{z}\left(t\right)\mu_{0}}{V\chi_{bead}}$$

By substituting Equation (11) into Equation (10), Equation (10) is achieved:

(12) $$Y\left(z,t\right)\frac{\partial Y(z,t)}{\partial z}=U\left(t\right)$$

Therefore, the final dynamic equations are considered as Equation (13):

(13) $$b_{z}\frac{\partial b_{z}}{\partial z}=\frac{\mu_{0}}{V\chi_{bead}}\cdot \frac{\left(1-bz^{-1}\right)\left(1+cz^{-1}+dz^{-2}\right)\left(1-ez^{-1}+fz^{-2}\right)}{\left(1-gz^{-1}+hz^{-2}\right)\left(1+iz^{-1}+jz^{-2}\right)\left(1-kz^{-1}+lz^{-2}\right)}\;\;a\;\;v\left(z\right)$$

where $b_{z}$ is the magnetic field in the direction of *z*, $\frac{\partial b_{z}}{\partial z}$ is the magnetic field gradient and v(z) is the velocity of the nanoparticle crossing the membrane. $\mu_{0},V$ and $\chi_{bead}$ are defined in Table 3. The problem is to ascertain the magnetic field and its gradients in order to cross the membrane in a defined time. Since the distance of crossing is constant and related to the membrane thickness, the suitable velocity is achieved by scaling the time as described in Equation (14):

(14) $$\left.\begin{array}{c} x_{membrane}=vt\\ x_{membrane}=v_{s}t_{s} \end{array}\right\}\to v_{s}=v\frac{t}{t_{s}}$$

Attaining suitable velocities in Equation (14) and substituting in Equation (13), a suitable range of velocities is obtained. Therefore, Equation (15) illustrates a suitable range for the magnetic field and its gradients:

(15) $$\left\{\begin{array}{c} b_{z}\frac{\partial b_{z}}{\partial z}=\overset{L_{\min}}{\overset{︷}{\frac{\mu_{0}}{V\chi_{bead}}\cdot \frac{\left(1-bz^{-1}\right)\left(1+cz^{-1}+dz^{-2}\right)\left(1-ez^{-1}+fz^{-2}\right)}{\left(1-gz^{-1}+hz^{-2}\right)\left(1+iz^{-1}+jz^{-2}\right)\left(1-kz^{-1}+lz^{-2}\right)}\;\;a\;\;v_{s_{\min}}\left(z\right)}}\\ b_{z}\frac{\partial b_{z}}{\partial z}=\overset{L_{\max}}{\overset{︷}{\frac{\mu_{0}}{V\chi_{bead}}\cdot \frac{\left(1-bz^{-1}\right)\left(1+cz^{-1}+dz^{-2}\right)\left(1-ez^{-1}+fz^{-2}\right)}{\left(1-gz^{-1}+hz^{-2}\right)\left(1+iz^{-1}+jz^{-2}\right)\left(1-kz^{-1}+lz^{-2}\right)}\;\;a\;\;v_{s_{\max}}\left(z\right)}} \end{array}\right.$$

and consequently:

(16) $$L_{\min}\leq b_{z}\frac{\partial b_{z}}{\partial z}\leq L_{\max}$$

Overall, MRI devices limit the application of the magnetic field and its gradients introduced in Equation (17):

(17) $$\left\{\begin{array}{c} 0\leq b_{z}\leq 2.4\left[T\right]\\ 0\leq \frac{\partial b_{z}}{\partial z}\leq 180\left[\frac{mT}{m}\right] \end{array}\right.$$

**Figure 10:**
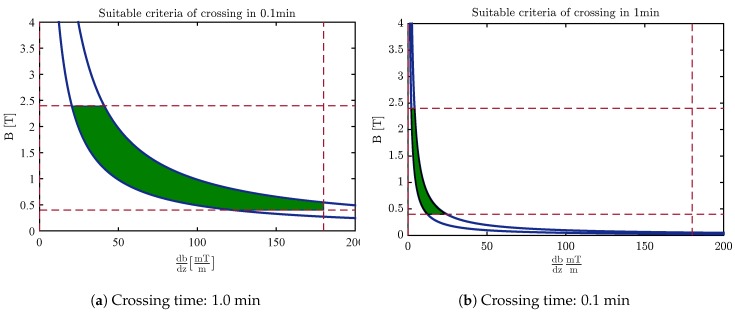
The desired area for selecting the magnetic field and magnetic gradients of fullerene.

**Figure 11:**
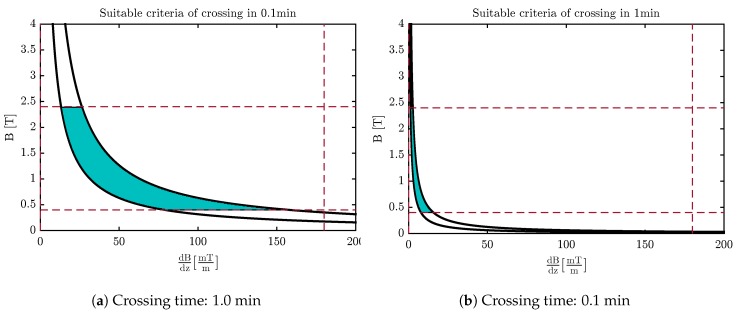
The desired area for selecting the magnetic field and magnetic gradients of the end-capped CNT.

Figure 10 and Figure 11 indicate an appropriate valid area of the magnetic field and its gradients based on the duration of crossing the membrane by applying these criteria along with contemplating the time of crossing. As perceived in this figure, the magnetic field and its gradient are situated at a lower value by increasing the time of crossing.

Finally, carbon nanocapsules can pierce the membrane without destroying its structure by using the optimal magnetic field. The nanoindentation process is similar for CNT and fullerene, although it seems that fullerene is the better choice since it causes less damage to the membrane structure (Figure 7) and covers a wider region in the desired area for selecting the magnetic field and its gradients’ free energy barrier to penetrate the phospholipid bilayer (Figure 10 and Figure 11).

## 5. Conclusions

Quite recently, CNTs have been considered as nanocarriers and have been extensively used for the possible nanoinjection of therapeutic agents into cells. In this study, nanotube and nanosphere structures have been modeled. The force needed to cross the membrane emerges through molecular dynamics simulation. In this simulation, the noninvasive forces for crossing at different velocities have been recorded, and the membrane frequency domain model has been applied to this set of data. By using the system identification method, a linear model has been fit to the data, and the relation between the forces and the velocities was expressed in the frequency domain. In this approach, the magnetic nanoparticle played the main role as an actuator, which is controlled in a precise way by changing the magnetic field. In order to design the suitable pathway, the magnetic field and membrane resistance have been solved inversely, and the desired magnetic field and magnetic gradients have been calculated. This nanosystem’s dynamics can be controlled by a non-invasive method of crossing the membrane. The magnetic profile and optimal magnetic field that result in the minimum work to cross the membrane have been determined. MRI devices, besides imaging, can provide the magnetic field by manual programming. The region defined for the magnetic field is supported practically by the typical MRI devices in the lab. By the selection of the range of magnetic fields and gradients, the crossing time would be adjusted automatically. The green and blue regions extracted from the simulation show the magnetic field and magnetic gradient needed for fullerene and CNT to cross the membrane in 0.1 min and 1 min, respectively. In our future work, the effect of the drug inside the CNT and its resistance inside the cell and blood circulation will be examined practically. This would be a new approach toward optimal controlled drug delivery to the cell.

## Figures and Tables

**Table 1:** Properties of two types of nanocapsules.

Nanocarrier Property	Fullerene	End-Capped CNT
Size	*R* = 2.41 nm	*R* = 2.41 nm and *l* = 7.40 nm
Number of atoms	720	2200
Number of insulin chains	4	4
Number of total atoms	88,328	121,488
Lipid size	$130\times 130\times 54{Å}^{3}$	$150\times 150\times 54{Å}^{3}$

**Table 2:** Transfer function parameter values.

Parameter	End-Capped CNT	Fullerene
*a*	$-2.83\times 10^{-10}$	$-4.60\times 10^{-10}$
*b*	−1.18	−1.07
*c*	+1.67	+1.80
*d*	+0.95	+1.05
*e*	−0.24	−0.11
*f*	+0.97	+1.06
*g*	−1.99	−1.99
*h*	+0.99	+0.99
*i*	+1.69	+1.73
*j*	+0.98	+0.97
*k*	−0.24	−0.09
*l*	+0.99	+0.97

**Table 3:** Parameter definitions and values.

Parameter	Definition	Value
$\mu_{0}$	The permeability of free space	$4\pi \times 10^{-7}$
*V*	Particle volume	$\frac{4}{3}\pi {r_{a}}^{3}$
$r_{a}$	Radius of the nanoparticle	2 nm
$\chi_{bead}$	Magnetic susceptibility	0.17
